# Unraveling RNA dynamical behavior of TPP riboswitches: a comparison between *Escherichia coli* and *Arabidopsis thaliana*

**DOI:** 10.1038/s41598-019-40875-1

**Published:** 2019-03-12

**Authors:** Deborah Antunes, Natasha Andressa Nogueira Jorge, Mauricio Garcia de Souza Costa, Fabio Passetti, Ernesto Raul Caffarena

**Affiliations:** 1 Computational Biophysics and Molecular Modeling Group. Scientific Computing Program (PROCC), Fundação Oswaldo Cruz, Rio de Janeiro, 21040-222 Brazil; 2 Laboratory of Functional Genomics and Bioinformatics, Oswaldo Cruz Institute, Fundação Oswaldo Cruz, Rio de Janeiro, 21040-360 Brazil; 3 Laboratory of Gene Expression Regulation, Carlos Chagas Institute, Fundação Oswaldo Cruz, Curitiba, 81350-010 Brazil

## Abstract

Riboswitches are RNA sensors that affect post-transcriptional processes through their ability to bind to small molecules. Thiamine pyrophosphate (TPP) riboswitch class is the most widespread riboswitch occurring in all three domains of life. Even though it controls different genes involved in the synthesis or transport of thiamine and its phosphorylated derivatives in bacteria, archaea, fungi, and plants, the TPP aptamer has a conserved structure. In this study, we aimed at understanding differences in the structural dynamics of TPP riboswitches from *Escherichia coli* and *Arabidopsis thaliana*, based on their crystallographic structures (TPPsw^ec^ and TPPsw^at^, respectively) and dynamics in aqueous solution, both in *apo* and *holo* states. A combination of Molecular Dynamics Simulations and Network Analysis empowered to find out slight differences in the dynamical behavior of TPP riboswitches, although relevant for their dynamics in bacteria and plants species. Our results suggest that distinct interactions in the microenvironment surrounding nucleotide U36 of TPPsw^ec^ (and U35 in TPPsw^at^) are related to different responses to TPP. The network analysis showed that minor structural differences in the aptamer enable enhanced intramolecular communication in the presence of TPP in TPPsw^ec^, but not in TPPsw^at^. TPP riboswitches of plants present subtler and slower regulation mechanisms than bacteria do.

## Introduction

Riboswitches are natural ribonucleic acid (RNA) sensors that affect post-transcriptional processes through their ability to bind to small molecules such as vitamins, amino acids and nucleotides^1,2^. Riboswitches are highly conserved and structured elements located in the untranslated regions (UTRs) or introns of pre-mRNAs and can be found in all the three domains of life^3–5^.

Riboswitches have been shown to modulate gene expression by influencing transcription, translation, alternative splicing and RNA stability^6–8^. Typically, genes regulated by a given riboswitch are involved in biosynthesis, catabolism, signaling or transport of a metabolite that binds to the riboswitch, creating feedback regulation mechanisms to control its adequate levels^9^. When the threshold of a metabolic pathway increases, binding of the metabolite to the Riboswitch leads to a negative feedback mechanism resulting in the suppression of the involved genes. Riboswitches display high specificity for the substrate, conferring efficiency to carry out their activity even in the presence of other similar metabolites^10^.

The structure of riboswitches consists of a two-domain set: the sensory and the regulatory domains. The sensory domain is the aptamer, whose sequence and structure are highly conserved. It acts as a receptor for particular metabolites, whereas the binding of small molecules are transduced to genetic regulatory signals by the expression platform localized adjacent to the aptamer^10^. This is achieved by a structural rearrangement, resulting in an immediate RNA conformational change that alters mRNA translation.

To date, the only riboswitch described in eukaryotes was the TPP riboswitch, since most of the studies have been carried out in prokaryotic organisms^11^. In bacteria, such as *Escherichia coli*, two functionally different TPP riboswitches were reported. The first one is located in the 5′UTR region of the *thiM* gene, where it controls gene expression at the translation level^11,12^, while the second guides both translation and transcription of 5′ UTR region of the *thiC* gene^11^.

TPP riboswitches have been found in 5′ UTRs regions of genes encoding thiamine biosynthetic enzymes in fungi^13,14^, and algae^15^, in which they promote alternatively spliced transcripts. Conversely, in all species of plants previously studied, the TPP riboswitch resides in the 3′ UTRs region of the *thiC* gene. This difference in mRNA localization suggests a unique mode of action for plant riboswitches^8,16^.

The TPP aptamer has sequence and structures highly conserved, independently if the gene is involved at different stages of the synthesis or transport processes of thiamine in bacteria, archaea, fungi, and plants. Rfam database^17^ has a total of 9180 TPP riboswitch sequences (Rfam accession RF00059) from distinct organisms, and the consensus secondary structure of these entries is highly conserved. Structurally, the TPP aptamer consists of five stems. The P1 stem is responsible for connecting the aptamer domain to the expression platform. Stems P2 and P3 are involved in binding of the TPP pyrimidine ring while stems P4 and P5 bind to the pyrophosphate group.

Most of the observed differences among species are found in the P3 stem region. For instance, bacteria and archaea commonly have a P3a stem^18^ not observed in eukaryotic riboswitches, whereas in eukaryotic organisms, the P3 stem is significantly variable in length, sequence, and base pairings^19^.

Despite the fact that both prokaryotic and eukaryotic TPP aptamers are structurally similar and play roles in the regulation of genes involved in thiamine biosynthesis, smaller structural differences may affect the way TPP binds and influences riboswitches in species of bacteria and plants. To analyze these differences, we performed a computational study using molecular dynamics simulations and correlation network analysis to compare the TPP riboswitch dynamical behavior in two of theses organisms. Our results suggest that distinct interactions in the microenvironment surrounding nucleotide U36 of TPPsw^ec^ (and U35 in TPPsw^at^) are related to different responses to TPP. We also showed that minor structural differences in TPP riboswitch made the regulatory mechanism of *A. thaliana* subtler and slower than *E. coli*.

## Results

### RNA content analysis

**Figure 1:**
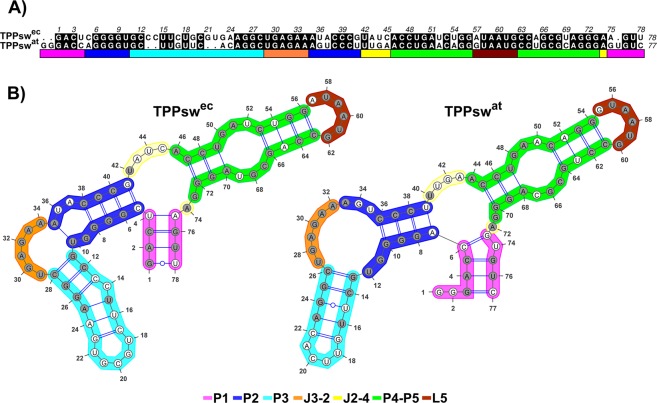
Sequence alignment and secondary structures of TPPsw^ec^ and TPPsw^at^. (**A**) Sequence alignment between TPPsw^ec^ and TPPsw^at^. Black filled positions of the alignment represent conserved residues. (**B**) Secondary structure of TPPsw^ec^ and TPPsw^at^. Conserved nucleotides were colored in grey. Stems, loops, and junctions were identified according to the figure legend caption.

Nucleotide sequence alignment between of *Escherichia coli* (TPPsw^ec^) and *Arabidopsis thaliana* (TPPsw^at^) revealed they have 68% of identity (Fig. 1A), along with highly conserved secondary structures (Fig. 1B). According to the SimTree server^20^, the secondary structure analysis indicated a normalized score of 0.68 in a scale from 0 to 1, where 1 indicates a perfect match and 0 no match at all. Both sequence and secondary structure share similar conservation.

A detailed inspection of the secondary structure content showed that the aptamers domains display identical J3-2 junctions and an equal number of pairings in the P1, P4, and P5 helices. On the other hand, the P3 helix was the least conserved substructure, presenting four nucleotides and an additional base pair in TPPsw^ec^. It is noteworthy that although the number of nucleotides in the P1 helix of TPPsw^ec^ is smaller than in TPPsw^at^, the number of base pairing remains the same, being four for each case. Differences can also be found in the J2-4 junction and P2 helix, in which an additional pairing in TPPsw^ec^ is formed. Altogether, the 2D riboswitch structures are highly conserved between species, although minor differences are observed mainly concerning the content of base pairings, being TPPsw^ec^ two pairings longer than TPPsw^at^.

**Figure 2:**
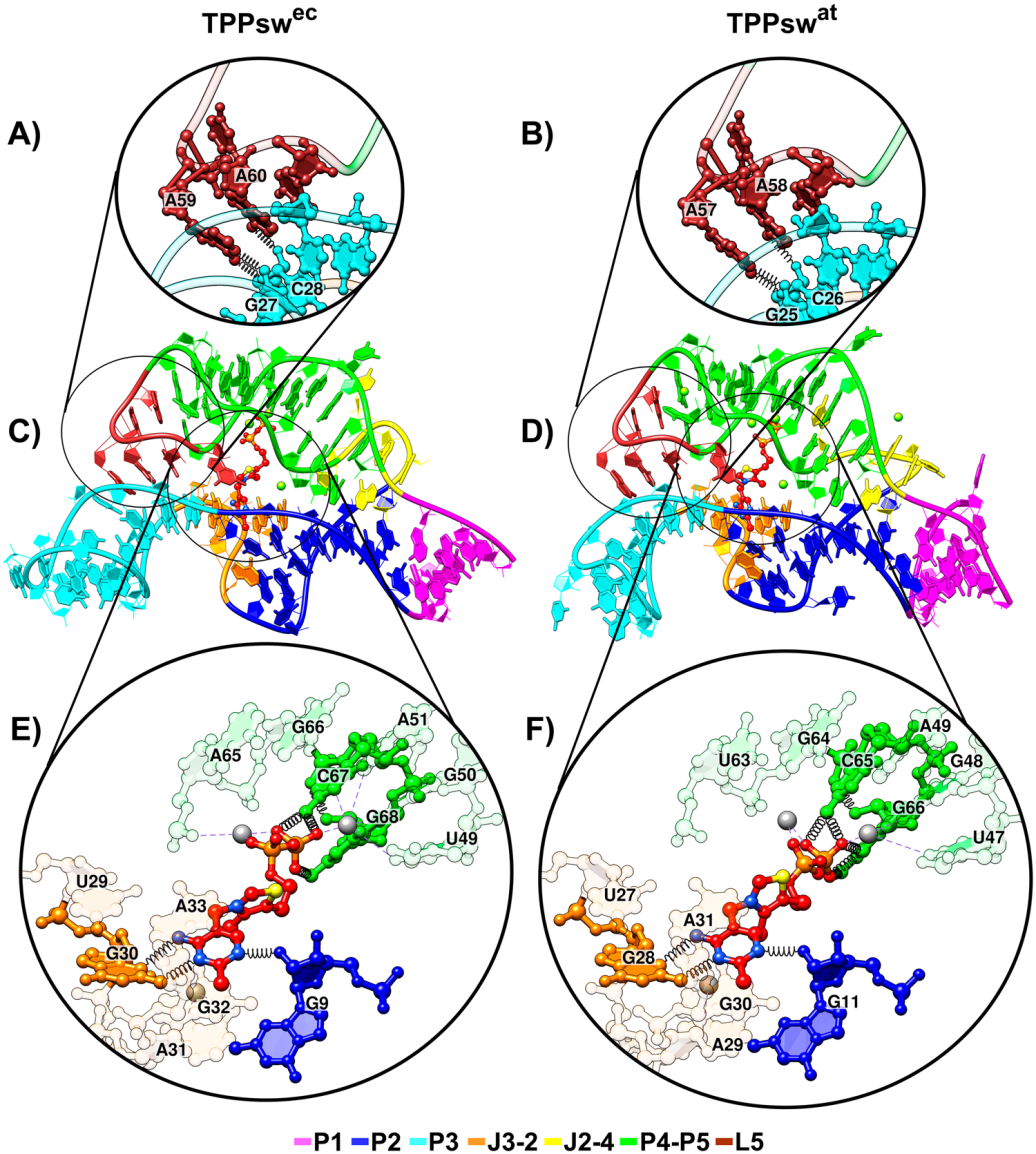
Tertiary structures of TPPsw^ec^ and TPPsw^at^. Cartoon and stick representation of P3-L5 substructures (**A**,**B**), whole aptamer (**C**,**D**), and TPP binding site (**E**,**F**). Stems, loops, and junctions are figure caption. Black springs and gray dashed lines indicate hydrogen bonds formation and interactions with the Mg^+2^ ion respectively (**A**,**B**,**E**,**F**). (**A**,**B**) Nucleotides A59(57) and A60(58) from loop L5 connect with residues G27(25) and C28(26) from the P3 minor groove. (**E**,**F**) The aminopyrimidine ring of TPP formed hydrogen bonds with G30(28) and the 2-OH′ of G9(11). Direct contacts to nonbridging oxygens of β-phosphate of TPP were also established via N4 of C67(65) and N1 of G68(66). All other pyrophosphate-RNA contacts were mediated through two Mg^2+^ ions (colored in grey).

Both prokaryotic and eukaryotic TPP aptamers share a notable common 3D structure and organization. The superposition between TPPsw^ec^ and TPPsw^at^ crystal structures resulted in heavy atoms root-mean-square deviation (RMSD) of approximately 0.63 Å (Fig. 2C,D). The insertion and relative position of TPP is also similar presenting a 0.69 Å RMSD. The aptamer consists of a switch helix (P1) and two sensor arms (P2/P3 and P4/P5), forming a long-range tertiary rearrangement capable of stabilizing the interaction between the L5 loop and the P3 stem (Fig. 2A,B). In both crystal structures, the P2/P3 arm helps accommodate the TPP pyrimidine ring while residues from the P4/P5 arm interact with the TPP pyrophosphate group (Fig. 2E,F).

### Global and local stability of the aptamer domain

**Figure 3:**
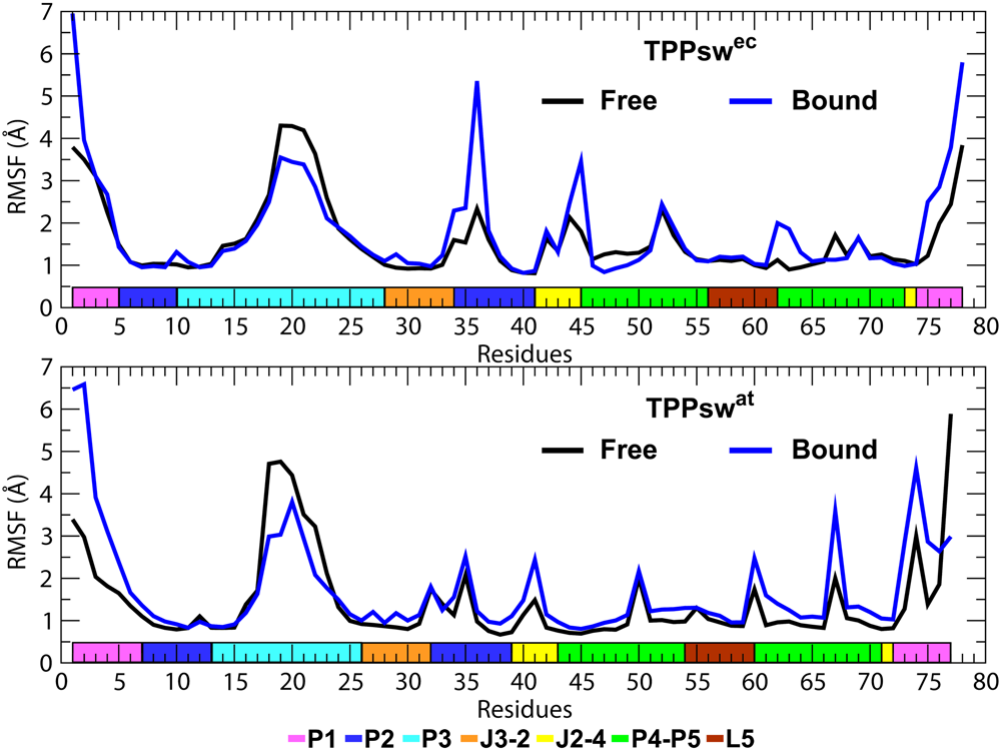
RMSF for the TPPsw^ec^ and TPPsw^at^ heavy atoms in free and bound forms. The aptamer substructures are depicted at the lower margin of the plots and colored according to the figure caption.

The structural stability of riboswitches in aqueous solution, in both *apo* and *holo* configurations, was evaluated by comparing the average RMSD (Table 1 and Supplementary Fig. S1) and root-mean-square fluctuation (RMSF) (Fig. 3) values calculated over the trajectory production stage, taking as references the structures obtained after equilibration.

**Table 1:** Root mean square deviations (Å) of free and bound states of TPPsw^ec^ and TPPsw^at^ as a whole and for substructures.

	Free (Å)		Bound (Å)	
	TPPsw^ec^	TPPsw^at^	TPPsw^ec^	TPPsw^at^
Whole	2.83 (0.45)	3.18 (0.34)	2.62 (0.41)	2.73 (0.37)
P1	5.91 (1.56)	4.97 (0.67)	6.44 (1.71)	5.22 (0.71)
P2	1.70 (0.33)	2.37 (0.40)	2.81 (0.80)	2.06 (0.27)
P3-L3	2.99 (0.66)	3.16 (0.48)	2.88 (0.58)	2.87 (0.50)
P4-P5	1.70 (0.25)	2.03 (0.17)	1.65 (0.27)	1.76 (0.35)
J3-2	1.37 (0.29)	1.78 (0.26)	1.85 (0.42)	1.94 (0.43)
J2-4	3.42 (0.38)	2.96 (0.40)	3.74 (0.26)	1.63 (0.61)
L5	1.43 (0.29)	1.41 (0.31)	1.53 (0.41)	1.52 (0.48)
TPP	—	—	1.38 (0.30)	1.41 (0.36)

The number in parenthesis indicates the RMSD standard deviation. “—” no RMSD values.

During Molecular Dynamics (MD) simulations, both *apo* and *holo* forms presented deviations around 3 Å. No noticeable differences have been observed between the RMSD values obtained for both TPPsw^ec^ and TPPsw^at^ upon TPP complexation. Indeed, both average RMSD values per region and the overall pattern of fluctuations were similar for all systems. These values are consistent with RMSD values seen in other studies reporting simulations of small RNA aptamers^21–24^ which ranged between 3–4 Å approximately.

The inspection at some particular regions of the RNA aptamer evidenced slight differences in their dynamical behavior. For example, the P1 region presented higher RMSD values than that of all other substructures in both species. Also, this region was more flexible in the *holo* forms. It is worth mentioning that the nucleotides composing the P1 helix are located in the terminal regions of the aptamer, showing broader movements during MD simulations. Unlike the P1 segment, the P3 helix displayed higher RMSD values in the *apo* forms. In addition, the comparison of the fluctuations between *apo* and *holo* riboswitches evidenced that TPP binding resulted in the stabilization of the P3 helix but contributed at the same time to the disruption of the P1 helix, according to the RMSF analysis (Fig. 3). Interestingly, the nucleotide U36, located in the P2 helix of TPPsw^ec^, displayed more significant fluctuations in the *holo* form than in the apo form (*holo*: 5.4 Å; *apo*: 2.3 Å) (Fig. 3). No significant changes were observed in the P2 substructure in *apo* and *holo* form of TPPsw^at^.

### Monitoring P3-L5 interaction

Despite being remotely located from the TPP binding site, the interaction between P3-L5 substructures is essential for metabolite binding^25,26^. The conformation of the P3-L5 region is kept in formation via two non-Watson-Crick (WC) base pairs. Nucleotides A59(57) and A60(58) from loop L5 connect with residues G27(25) and C28(26) from the P3 minor groove (Fig. 2A,B)^27^. Numbers in parenthesis indicate the nucleotide position in the TPPsw^at^ systems.

By monitoring the formation of non-WC hydrogen bonds between P3 and L5 we observed that, notably, the G27(25)·A59(57) interaction in *apo* TPPsw^ec^ was the less stable with 68% occupancy during the simulation (Table 2). Upon TPP binding, the occupancy increased to 74% while no corresponding trend was observed upon comparison of the *apo* and *holo* TPPsw^at^ systems (presenting ~81% occupancy in both cases). Also, the C28(26)·A60(58) was more stable and presented occupancies higher than 84% in all systems. In addition, the base pair G27·A59 of TPPsw^ec^ appears to be influenced by the presence of the ligand, which contributed to its stabilization.

**Table 2:** Occupancy of hydrogen bonds between non-Watson-Crick pairs involved in the P3-L5 interaction of free and bound states of TPPsw^ec^ and TPPsw^at^.

Nucleotides		frames (%)			
		Free		Bound	
P3	L5	TPPsw^ec^	TPPsw^at^	TPPsw^ec^	TPPsw^at^
G27(25)	A59(57)	68	82	74	81
C28(26)	A60(58)	93	89	89	84

### TPP-RNA interaction

**Figure 4:**
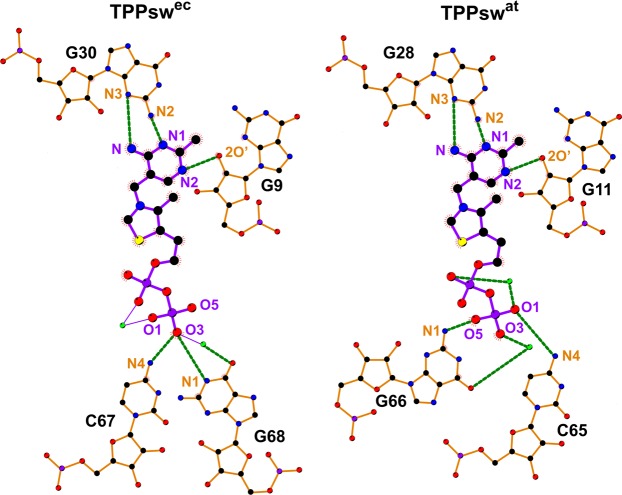
2D representation of the RNA-TPP interaction of TPPsw^ec^ and TPPsw^at^ crystallographic structures. TPP, RNA and Mg^+2^ ion are represented in purple, orange and green, respectively. C, N, O, P and S atoms are colored in black, blue, red, purple, and yellow, respectively. Green dashed lines indicate hydrogen bonds formation. The figure was generated using LigPlot+^57^.

To verify the stability of TPP-RNA complexes during MD simulations, we calculated the H-bonds occupancy and the average distances between pairs of atoms involved in key interactions (Table 3). We confirmed the existence of similar interactions in all systems. The aminopyrimidine ring of TPP formed hydrogen bonds with N2 and N3 of G30(28) and the 2-OH′ of G9(11). Direct contacts to non-bridging oxygens of TPP β-phosphate were also formed via N4 of C67(65) and N1 of G68(66). All other pyrophosphate-RNA contacts were mediated through the two Mg^2+^ ions^26,27^ (Fig. 4).

**Table 3:** Occupancy and distance of RNA-TPP interaction. The number in parenthesis indicates the distance standard deviation. “—” no RMSD values.

Atoms		Occupancy (%)		Distance (Å)	
RNA	TPP	TPPsw^ec^	TPPsw^at^	TPPsw^ec^ (SD)	TPPsw^at^ (SD)
G9(11) – 2O′	TPP – N2	56	26	2.86 (0.19)	3.03 (0.35)
G30(28) – N2	TPP – N1	91	90	2.99 (0.14)	3.06 (0.17)
G30(28) – N3	TPP – N	64	70	3.08 (0.17)	3.02 (0.14)
C67(65) – N4	TPP – O1	41	0	3.05 (0.40)	5.18 (0.97)
C67(65) – N4	TPP – O3	3	10	4.02 (0.33)	4.67 (0.77)
C67(65) – N4	TPP – O5	1	35	4.70 (0.70)	4.08 (1.07)
G68(66) – N1	TPP – O1	6	0	3.71 (0.51)	4.17 (0.29)
G68(66) – N1	TPP – O3	0	0	3.31 (0.25)	3.24 (0.31)
G68(66) – N1	TPP – O5	30	26	3.64 (0.62)	3.65 (0.41)
Mg^2+^(1)	Mg^2+^(2)	—	—	6.58 (0.97)	3.35 (1.13)
Mg^2+^(1)	TPP – O3	—	—	1.85 (0.04)	1.89 (0.06)
Mg^2+^(2)	TPP – O1	—	—	3.38 (1.53)	1.90 (0.06)

Analysis of the distributions of the total number of RNA-TPP hydrogen bonds revealed that most TPPsw^ec^ formed more interactions than TPPsw^at^ (Supplementary Fig. S2). The number of sampled structures presenting four or more hydrogen bonds was consistently higher in the TPPsw^ec^ system than in TPPsw^at^. The occupancy of the two hydrogen bonds formed between G30(28) and TPP was similar for both systems. Curiously, the occupancy of G9(11) for TPPsw^at^ was less than a half (26%) than for TPPsw^ec^ (56%).

In the *holo* systems, the Mg^2+^(1) ion remained closer than 1.90 Å from O3 β-phosphate of TPP, interacting inespecifically with RNA. In TPPsw^at^, the magnesium atom Mg^2+^(2) got closer to Mg^2+^(1) (3.35 ± 1.13 Å) and interacted strongly with O1 oxygen atom from TPP (1.90 ± 0.06 Å), leaving the O5 oxygen atom from β-phosphate available to interact with RNA, with consequent formation of a bifurcated H-bond. The O5 oxygen atom from TPP interacted with C65 e G66, with hydrogen bond occupancies of 35% and 26%, respectively.

Each TPPsw^ec^ nucleotide involved in pyrophosphate recognition formed more than one hydrogen bond because they interacted with other oxygen atoms from TPP, being C67·TPP–O1 and G68·TPP–O5 examples of these interactions, with occupancies of 41% and 30%, respectively (Table 3).

### Principal component analysis suggests different dominant motions in *apo* and *holo* states

To identify statistically relevant motions of TPP riboswitches in solution, we performed principal component analysis (PCA) on the snapshots obtained from the MD trajectories. Overall, the first three components (named PC1, PC2, and PC3) captured the dominant motions, presenting the highest contributions to total fluctuations. The first three PCs accounted for 54.9% and 52.6% of the overall variance in *apo* TPPsw^ec^ and TPPsw^at^, respectively. In the *holo* states, the contribution of the first three PCs was slightly higher: 61.7% and 58.7% for TPPsw^ec^ and TPPsw^at^, respectively.

**Figure 5:**
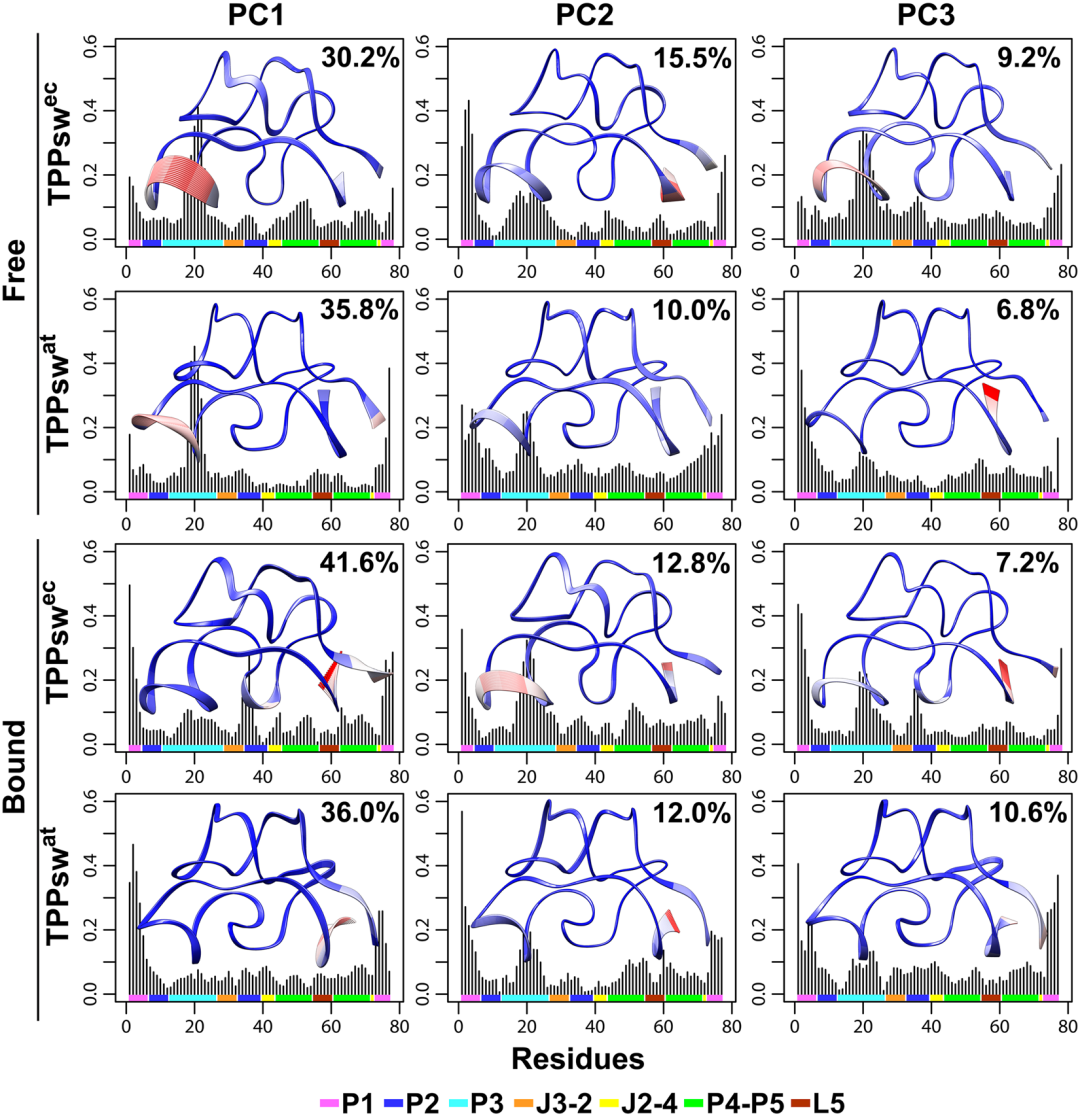
RMSF contributed by the first three principal components. The fractional contribution of each PC to the overall variance is shown in the top right part of each graph. Interpolated structures obtained by displacements along each vector are displayed within each plot. Blue indicates overlapping regions with little or no motion. Red areas represent mobile regions. The secondary structure elements are given at the lower margin of the plots and colored according to the figure caption.

We compared the projections of the trajectories onto the subspace spanned by the first three principal components. RMSFs and structural projections along the three PCs showed that the four systems displayed a uniform and overlapping PC subspace (Fig. 5).

Inspection of the atomic fluctuations along PC1 revealed substantial higher flexibility of the P3 helix in the *apo* systems, confirming its stabilization by ligand binding. However, this region presented higher fluctuations along PC2 in the *holo* state in the TPPsw^ec^ system, but not in TPPsw^at^. Therefore, the stabilization of the most statistical relevant motions of P3 helix inducted by ligand binding was undoubtedly more pronounced in TPPswat, as lower fluctuations were noticed along both principal components. Furthermore, TPP binding resulted in the augmented flexibility of the P1 helix. In the *holo* TPPsw^ec^, the ligand promoted an increase in the flexibility of nucleotides 34–37 of the P2 helix that was unseen in TPPsw^at^.

### Correlation network analysis reveals distinct responses to ligand binding in TPPsw^ec^ and TPPsw^at^

**Figure 6:**
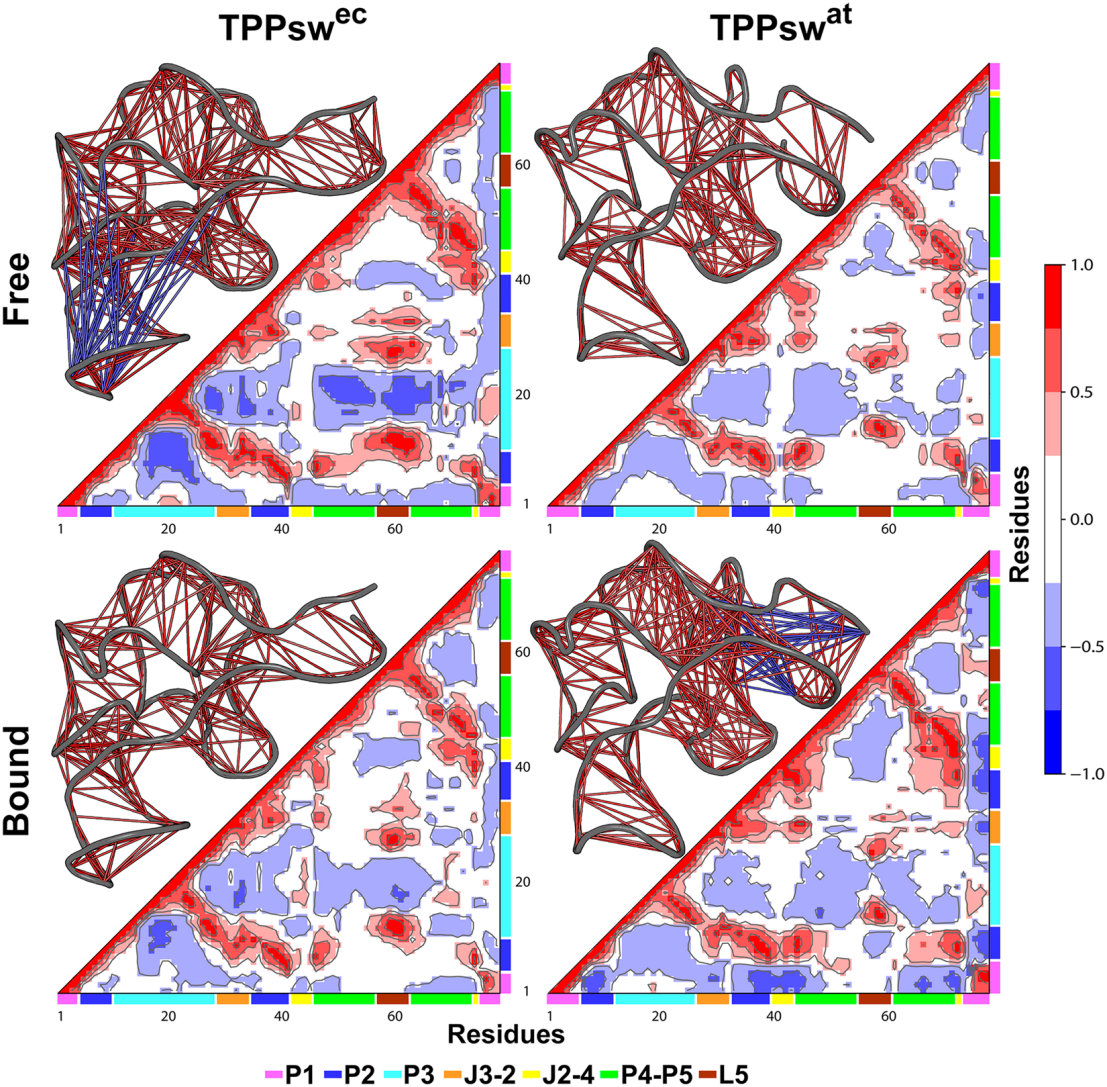
DCCMs of free and bound states of TPPsw^ec^ and TPPsw^at^. Next to each matrix, the corresponding 3D structures with lines connecting pairs of correlated residues are shown. For clarity sake, only the pairs presenting (|C_ij_|) > 0.6. are represented.

We analyzed the correlations between pairs of nucleotides to investigate how TPP binding affects the dynamic couplings in TPPsw^ec^ and TPPsw^a^. We calculated the dynamic cross-correlation matrices (DCCM) for each simulated system, as described in the methods section. A similar cross-correlation pattern was observed for both systems in their *apo* states. However, both the extent of regions displaying anticorrelations and their magnitudes were greater in TPPsw^ec^, mainly in the P3–P4-P5–L5 region (Fig. 6).

It was observed that TPP binding stabilized the P3-L5 interaction in TPPsw^ec^, as evidenced by weaker anticorrelations, which are likely to be associated with the separation of these regions in the *apo* state. This result is in line with PCA revealing increased stability of P3 motions in the holo state (Fig. 5). Interestingly, no similar trend was observed in TPPsw^at^. In TPPsw^at^ system, TPP binding resulted in increased anticorrelations at the P1-P2 helices, indicating a possible destabilization of interactions that ultimately resulted in a larger separation between them. In contrast, the dynamic coupling pattern in this region was not altered in TPPsw^ec^.

**Figure 7:**
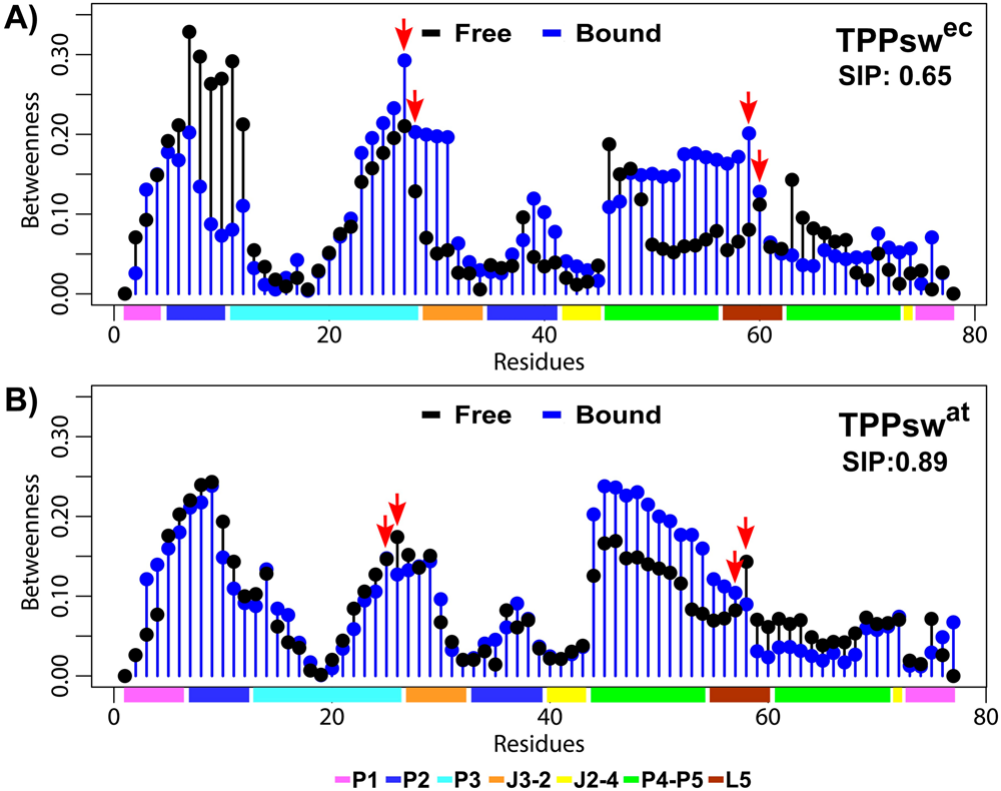
Betweenness centrality of the node for each residue of free and bound states. (**A**) TPPsw^ec^ systems. (**B**) TPPsw^at^ systems. Red arrows indicate residues G27(25)–A59(57) involved between P3-L5 interaction. Square Inner Product (SIP) is shown above each graph.

Next, we performed a correlation network analysis by constructing weighted graphs in which each residue was represented by a single node, and the weight of the connection between pairs of nodes was proportional to their respective previously calculated correlation coefficients. To quantify the relative importance of each residue in the network, we computed the betweenness centrality *per* nucleotide for each simulated system (Fig. 7). This metric is used to identify critical communication nodes over the network. Residues presenting high betweenness values are considered “bottlenecks” of information as they are found in the shortest communication paths^28^.

We calculated the square inner product (SIP) to compare the overall similarity of the betweenness centrality profiles calculated for the *apo* and *holo* states. According to this analysis, high SIP values are associated with weak modulation of intramolecular communication as a consequence of the TPP binding. We obtained a higher SIP for TPPsw^at^ (0.89) than for TPPsw^ec^ (0.65), thus reinforcing more noticeable TPP related effects in TPPsw^ec^ systems. In both *holo* systems, we noted increased centrality values at the P4-P5 helices. Interestingly, the nucleotides G27, C28, and A59 were critical for P3–L5 interaction and displayed higher betweenness values in *holo* TPPsw^ec^, indicating that ligand binding favors an efficient communication through these nucleotides (Fig. 7). This feature was not observed in TPPsw^at^, in which C26 and A58 centrality values were higher in the *apo* state.

### Communication pathways between P3-L5

**Figure 8:**
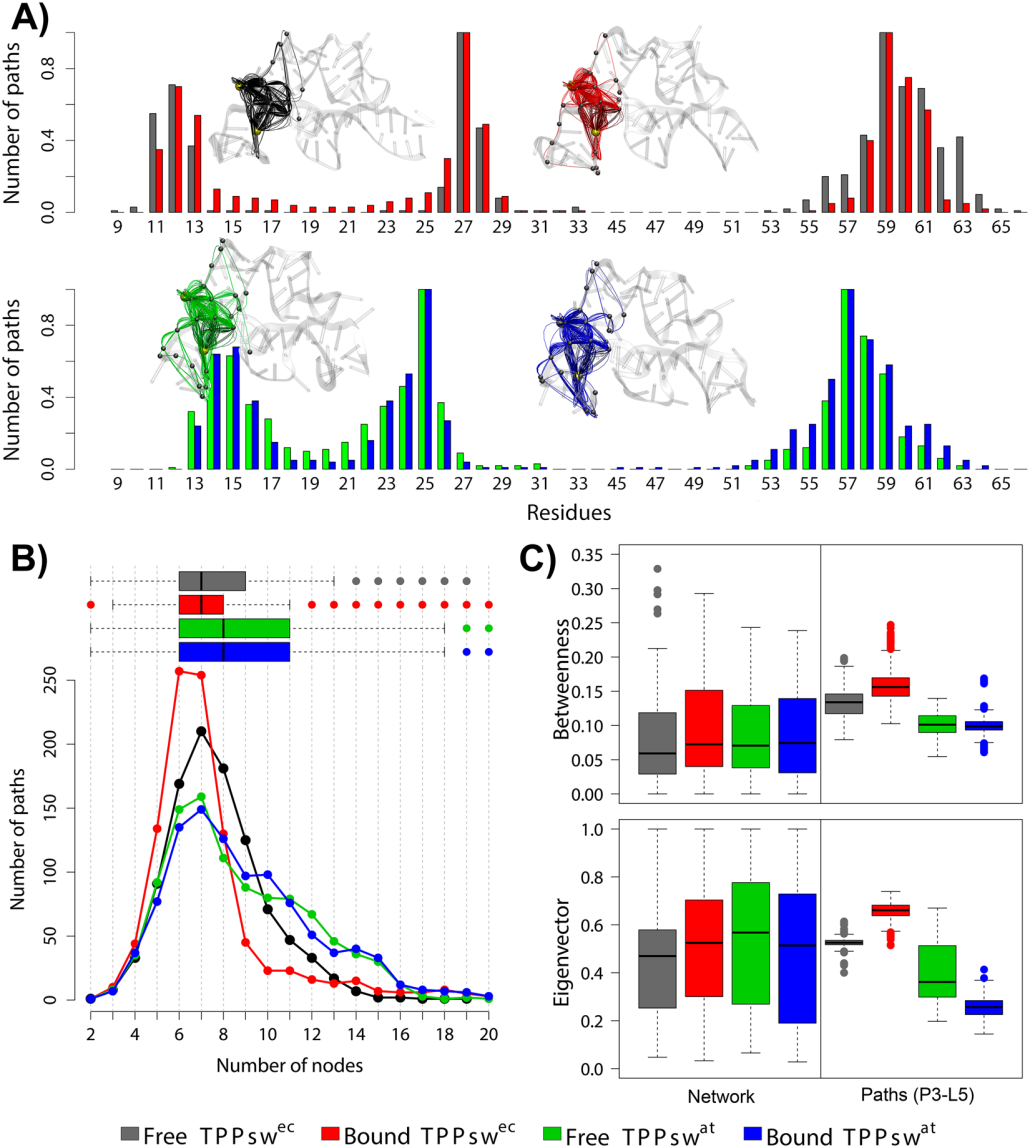
Shortest communication paths connecting G27(25) and A59(57) residues of free and bound TPPsw^ec^ and TPPsw^at^. (**A**) Normalized node degeneracy graph and visualization of sub-optimal paths in a correlation network. (**B**) Number of nodes per path. (**C**) Boxplot of betweenness and eigenvector centrality of the paths compared to the one corresponding to the complete network. Each system was colored according to the figure caption.

To get a deeper understanding about the critical residues governing P3-L5 interactions, we computed the 1000 shortest paths between G27(25) [located in P3] and A59(57) [located in L5] (Fig. 8). The normalized node degeneracy metric reveals the percentage of paths accessing each node. We observed for all systems conserved critical residues for communication with degeneracy values > 0.35. While the majority of these residues belongs to L5 (U58(56), A60(58) and G61(59)), two of them (C12(14) and C28(26)) are located in P3 (Fig. 8A).

The distribution of node degeneracies obtained for TPPsw^ec^ was narrower at the P3 region in the *holo* state, indicating that TPP binding restricted the presence of a few residues in the shortest paths. Whereas a larger number of residues are accessed in the *apo* state, the shortest paths were mostly formed by nucleotides 59–61 in the *holo* state. In contrast, the distributions obtained for both TPPsw^at^ states were very similar (Fig. 8A).

We calculated the number of nodes per path to further evaluate and characterize the influence of TPP binding on P3-L5 interactions (Fig. 8B). This analysis was based on the hypothesis that communication involving fewer nodes along the pathway is likely to be more efficient. Indeed, in *holo* TPPsw^ec^, the P3-L5 communication required fewer nodes (Fig. 8B). Interestingly, while TPP binding did not modify the global distributions of betweenness centralities obtained for both species (Fig. 8C left boxes), opposing trends were perceived concerning the average betweenness centrality calculated for the residues participating in shortest paths (Fig. 8C right boxes). However, TPPsw^ec^ TPP binding resulted in increased centralities for the residues involved in shortest paths, leading to a slight decrease in average betweenness in TPPsw^at^.

To support this analysis, we computed the eigenvector centralities for the overall network and the shortest paths (Fig. 8C lower boxes). Again, TPP binding resulted in higher centrality in the shortest paths only for TPPsw^ec^. The selection of specific P3 residues imposed by ligand binding in TPPsw^ec^ resulted in a stronger communication along pathways accessing a selection of neighboring residues with high eigenvector centrality. In agreement with our previous analysis (Figs 6–8), a corresponding effect was not observed in TPPsw^at^, which strongly suggests the weaker influence of TPP for an effective communication.

## Discussion

Plant and bacterial TPP aptamers share similar core structures and bind to the same ligand. However, minor structural and dynamical differences between them could be found, specially concerning the behavior of P3 helix. Bacteria and archaea commonly have a P3a stem^18^, which is not observed in eukaryotic riboswitches. The eukaryotic P3 stem is significantly variable in length, sequence, and base pairings^19^. Particularly in plants, the length of distal P3 extension varies among TPP aptamer representatives of the same species, as observed in *Physcomitrella patens*^8^. The P3 distal portion is not required for ligand binding of L5-P3 interaction^5,29^, but might act as an anchor for the aptamer as it was already pointed out by Anthony *et al*.^30^. Also, the authors claim that the correct folding could help in the competition with other RNA structures with different regulation mechanisms.

However, despite that the P3 stem is significantly variable in length in plants, the TPP aptamer is structurally stable. This stability might lead to slower arm movement than the observed in the helix arm of *E. coli* TPP riboswitch^31^. Cross-correlation analysis corroborated this hypothesis because stronger negative correlations were noticed in the *apo* TPPsw^ec^ involving substructures P3–P4-P5–L5 (Fig. 6). Our findings also suggest that communication pathways between P3-L5 may be different in *E. coli* and *A. thaliana*. The communication between P3-L5 in TPPsw^ec^ can be very efficient in the *holo* state, while in TPPsw^at^ the corresponding effect was weakened, thus suggesting a slower response to TPP binding in plants than in bacteria (Fig. 8).

The *A. thaliana* crystallographic structure used as starting point for our simulations contains a shortened P3 stem formed by 14 nucleotides. On the other hand, the corresponding structure in *E. coli* is composed of 18 nucleotides. The P3 helix of TPPsw^at^, although smaller than the one in TPPsw^ec^, showed no significant modifications in the presence of the ligand, indicating that the size of P3 can be oblivious to plants and its influence about slow folding can be negligible.

Guedich *et al*. wondered whether the slow TPPsw^at^ folding would be related to a single nucleotide. The authors concluded that U35, located on the P2 helix, is crucial for shaping a TPP-binding competent riboswitch^32^. In our analysis, the equivalent pyrimidine nucleotide in TPPsw^ec^ is U36 (Fig. 1B). The magnitude of the fluctuations at this position was 2-fold higher in the *holo* state than in the *apo* TPPsw^ec^ state (Fig. 3). In contrast, in the TPPsw^at^ system, similar fluctuations were perceived regardless of a ligand binding. Furthermore, PCA data also supported these outcomes by showing that the segment 34–37 of the *holo* TPPsw^ec^ displayed the most significant motion amplitude along PC1 (Fig. 5).

Grounded on these findings, we hypothesize that different interactions found in the microenvironment surrounding nucleotide U36 of TPPsw^ec^ (and U35 in TPPsw^at^) are related to different TPP responses. In TPPsw^ec^, this nucleotide is neighbored at 3′ by a non-canonical A37-G9 base pair. A similar context is observed for U35 in TPPsw^at^, which is delimited by a non-canonical G34-G11 base pair but on 5′ instead. Nucleotides G9 and G11 of TPPsw^ec^ and TPPsw^at^, respectively, form hydrogen bonds with the aminopyrimidine ring of TPP. Interestingly, our simulations have shown that hydrogen bond occupancy between G9(11) and N2 of TPP was less than a half for TPPsw^at^ (25.67%) than for TPPsw^ec^ (55.58%). This suggests that slight differences in the environment may directly interfere the TPP-aptamer interaction stability.

Finally, TPP riboswitches of *Arabidopsis thaliana* present subtler and slower regulation mechanisms than *Escherichia coli*^30–32^. Here, we have shown through molecular dynamics simulations and networking analysis that minor structural differences in the aptamer enable enhanced intramolecular communication in the presence of TPP in TPPsw^ec^, but not in TPPsw^at^. Weaker responses to changes in the TPP concentration may be related to the autotrophic mode of nutrition, which demands the endogenous synthesis of thiamine. Unlike in plants, bacteria can grow under thiamine-rich conditions allowing them to satisfy their full demand for compounds like thiamine exogenously^8^. Taken together, our results provide new insights into RNA behavior of TPP riboswitch, which may have adapted in a different way to the different metabolic demands of each group of organisms to accomplish distinct TPP binding modulation.

## Materials and Methods

### Analysis of crystallographic structures

For this study, TPP riboswitch 3D-structures of *Escherichia coli* (TPPsw^ec^) and *Arabidopsis thaliana* (TPPsw^at^) obtained by X-ray crystallography were selected from the Protein Data Bank (PDB)^33^. Both structures are bound to TPP and present high-resolution crystal, of 2.05 Å (PDB ID: 2GDI^27^) and 2.25 Å (PDB ID: 3D2G^29^), for TPPsw^ec^ and TPPsw^at^, respectively. Corresponding sequence and secondary structures information were also taken from the PDB files and analyzed with 3DNA software suite^34^. TPPsw^ec^ and TPPsw^at^ sequences were then aligned using the SARA-Coffee mode of T-Coffee program^35^, and figures of sequence alignment were rendered using ALINE^36^. The secondary structure calculated on the basis of the PDB files of the crystallographic structures was compared through the SimTree server^20^. SimTree returns a similarity score and a mapping between the similar regions of the two structures in dot-bracket notation. Graphical representation of 2D and 3D structures were generated using VARNA^37^ and UCSF Chimera, respectively.

### Molecular dynamics simulations

Molecular dynamics (MD) simulations were carried out using the GROMACS version 5.1.2 package^38^, and RNA interactions were represented using the amber *14sb*^39^ force field with *parmbsc0* + *chiOL3*^40^. Bonded and Lennard-Jones molecular parameters for TPP have been obtained using the Generalized Amber force field (GAFF)^41^ and AM1-BCC^42^ tools while atomic partial charges were added using ANTECHAMBER^43^. ACPYPE^44^ program was employed to create a GROMACS compatible topology file. Electrostatic interactions were treated using the particle mesh Ewald (PME) algorithm with a cut-off of 10 Å.

MD trajectories were monitored to investigate possible differences in the dynamical behavior between *apo* and *holo* TPPsw^ec^ and TPPsw^at^. In the *apo* systems, TPP was removed from the X-ray crystal structure and replaced with solvent water. Two initially positioned magnesium ions in the crystal structure were kept in both the *apo* and *holo* systems and also contributed to neutralize the systems. These ions are essential for ligand binding and confer stability to the riboswitch as well^45^. Each system was simulated under periodic boundary conditions in a triclinic box whose dimensions were automatically defined considering a distance of 1 nm from the outermost RNA atoms in all cartesian directions. The simulation box was filled with TIP3P water molecules^46^.

Simulations were performed in three stages: *(i)* Energy minimization, *(ii)* thermalization and equilibration, and *(iii)* trajectory production.

Energy minimization procedure was performed through 5000 steps and a gradient tolerance <1.0 kJ mol^−1^ nm^−1^ of the steepest descent and conjugate-gradient algorithms. These steps were carried out with heavy atom restraints by applying a harmonic potential with a force constant of 1000 kJ mol^−1^ nm^−2^ for the steepest descent algorithm. Applications of the conjugate-gradient algorithm do not allow the application of restraints.

In the second phase, starting atomic velocities were assigned to all atoms of the system using a Maxwell-Boltzmann distribution, corresponding to an initial temperature of 20 K. Then, the systems were gradually heated up to 300 K over 500 picoseconds (ps) utilizing the Langevin thermostat. During this stage, all heavy atoms were harmonically restrained by applying a constant force of 1000 kJ mol^−1^ nm^−2^.

Systems were subsequently equilibrated during twenty successive 100 ps long equilibration simulations where position restraints progressively approached zero. After this period, the systems were simulated with no restraints all at 300 K for 1 µs (trajectory production). All simulations were performed in the NPT ensemble. The V-rescale thermostat and Berendsen barostat were used for temperature (300 K) and pressure control (1 atm), respectively.

### Trajectory analysis

As in the trajectory analysis, we were interested only in the structural aspects of the systems, regardless the temporal correlation. Two independent MD simulations were concatenated, and trajectory analyses were conducted for crystal structures systems.

To investigate structural changes of the TPP aptamers, root-mean-square deviation (RMSD) values were calculated separately for the whole RNA and its substructures after fitting to their respective parts, taking the initial structure of the production dynamics as a reference. Hydrogen bond formation was defined using a geometric criterion with VMD software. It was considered a hit when the distance between two polar heavy atoms, with at least one hydrogen atom attached, was less than 3.5 Å using a D-Ĥ-A angle cutoff of 30°. Motif Identifier for Nucleic acids Trajectory (MINT)^47^ program was used to evaluate the number of Watson–Crick (WC)-edge and non-WC-edge hydrogen bonds (and their sum) per nucleotide throughout the simulations.

### Principal components analysis

The study of large-scale domain motions is essential for characterizing the conformational dynamics of macromolecules. Functional *motions* are usually described by a few numbers of degrees of freedom that can be calculated using principal component analysis (PCA)^48,49^. PCA analysis was carried out for all systems using Bio3D^50^ library as implemented in R^51^. Rotational and translational motions were removed before calculation of the covariance matrix by least-squares superposition to the corresponding average structures. The 3 N × 3 N covariance matrices of C5′ atomic positions (Cartesian coordinates) were then calculated for each state. The conformations explored during the MD simulations were applied using hierarchical clustering in R (*hclust*) with the *complete* linkage method based on the PC1-PC2 subspace, where PC1 and PC2 denote the projections onto the two first eigenvectors.

### Correlation network analysis

The cross-correlation and network analyses were carried out using the Bio3D and the *igraph* R packages^52^. Initially, the dynamic cross-correlation matrices (DCCM) were calculated separately for each simulation using as inputs the corresponding MD trajectory superimposed onto the initial structure. Then, each group of two matrices per riboswitch state was utilized to obtain a consensus matrix. A proximity/contact map filter was applied in the construction of the correlation network for residues that remained within 4.5 Å from one another for at least 75% of simulation time. Briefly, graphs were obtained considering C5′ atoms as nodes and the connection between nodes *i* and *j* were weighted using the absolute values of cross-correlations (*C*_*(i,j)*_) coefficients (Equation 1):

1 $${w}_{(i,j)}=-\,\mathrm{log}(|{C}_{(i,j)}|).$$

We also calculated the relative importance of each node for communication using centrality measures. According to the definition of the betweenness centrality (Equation 2), the relevance of a given node is defined by its presence in shortest communication paths connecting nodes over the entire network^53^.

2 $${c}_{B}(n)=\sum _{i\ne j\ne k\in N}\frac{\sigma (i,j|n)}{\sigma (i,j)}$$

According to the above equation, the betweenness centrality of a node depends on the total number of the shortest paths between nodes *i* and *j* that pass-through *n* ($\sigma (i,j|n)$ and $\sigma (i,j)$, which is the total number of shortest paths between nodes *i* and *j* (regardless of whether they cross or not through *n*).

Another measure of centrality can be given by the eigenvector (Equation 3) that accounts for the global relevance of each residue based on the connections with neighboring nodes. In other words, nodes with high eigenvector centrality are those connected to other central residues^54^.

3 $${c}_{Ei}(v)=\alpha \sum _{\{u,v\}\in E}{c}_{Ei}(u)$$

The vector ${c}_{Ei}={({c}_{Ei}(1),\ldots ,{c}_{Ei}({N}_{v}))}^{T}$ is obtained as a solution to the eigenvalue problem $A{c}_{Ei}={\alpha }^{-1}{c}_{Ei}$, where *A* is the adjacency matrix for the network graph *G*. More mathematical details can be found at Kolaczyk^54^.

The Square inner product (SIP) (Equation 4) was used to compare the overall similarity of the centrality profiles calculated for the systems. It varies between 0 and 1 and is defined as

4 $$SIP=\frac{{({w}_{A}^{T}{w}_{B})}^{2}}{({w}_{A}^{T}{w}_{A})({w}_{B}^{T}{w}_{B})}$$

where *w*_*A*_ and *w*_*B*_ are *N-*length vectors containing the fluctuation value for each atom in proteins A and B, respectively^55^.

The Yen’s algorithm^56^ was used to calculate the shortest pathways connecting two nodes in the network. Path lengths are defined as the sum of the edge weights connecting a pair of nodes in a given pathway. The first 1000 shortest paths were collected and employed to calculate the node degeneracy value, which represents the percentage of pathways from the overall ensemble in which a given node is present.

## Supplementary information

Supplementary Figures
