# Supplementary material for: Unraveling RNA dynamical behavior of TPP riboswitches: a comparison between Escherichia coli and Arabidopsis thaliana
Source: Sci Rep. 2019 Mar 12;9:4197. doi: 10.1038/s41598-019-40875-1 (PMC6414600; doi:10.1038/s41598-019-40875-1)
Supplement: Supplementary file 1 — Supplementary Figures [file 41598_2019_40875_MOESM1_ESM.docx]

**<Supplementary Information>**

**Unraveling RNA dynamical behavior of TPP riboswitches: a comparison between *Escherichia coli* and *Arabidopsis thaliana***

Deborah Antunes^1^, Natasha Andressa Nogueira Jorge^2^, Mauricio Garcia de Souza Costa^1^, Fabio Passetti^2,3^ and Ernesto Raul Caffarena^1,*^

^1^Computational Biophysics and Molecular Modeling Group. Scientific Computing Program (PROCC), Fundação Oswaldo Cruz, Manguinhos, Rio de Janeiro 21040-222, Brazil

^2^Laboratory of Functional Genomics and Bioinformatics, Oswaldo Cruz Institute, Fundação Oswaldo Cruz, Rio de Janeiro 21040-360, Brazil

^3^ Laboratory of Gene Expression Regulation, Carlos Chagas Institute, Fundação Oswaldo Cruz, Curitiba 81350-010, Brazil

*Correspondence

Correspondence and requests for materials should be addressed to:

E.R.C. (ernesto.caffarena@fiocruz.br)


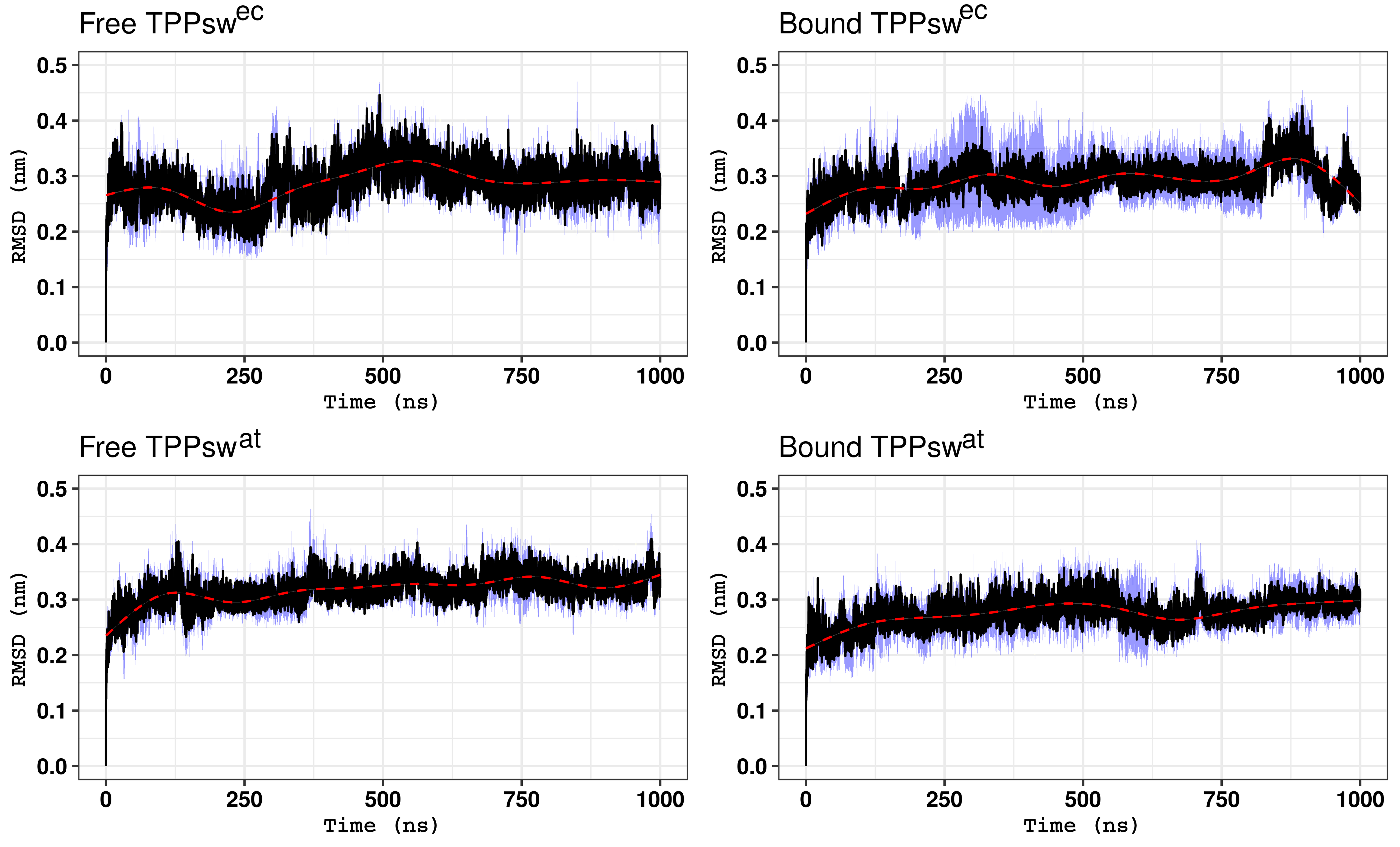


**Supplementary Figure S1.** RMSD of the TPPsw^ec^ and TPPsw^at^ in free and bound systems. Mean (black lines), confidence interval (lines smooth blue) and trend lines (red dashed line) of the replicates are displayed.


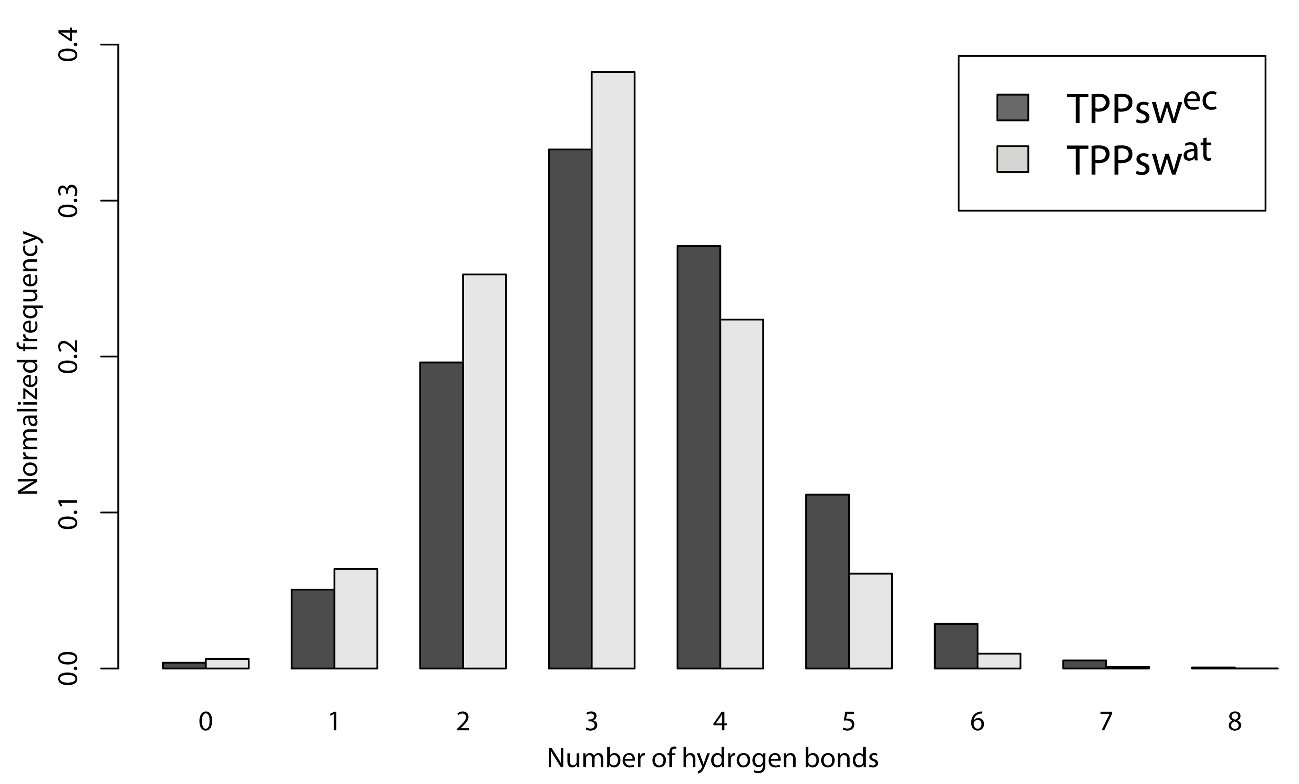


**Supplementary Figure S2.** Distribution of the number of hydrogen bonds formed between RNA and TPP of TPPsw^ec^ and TPPsw^at^ systems. Bars were normalized by hydrogen bonds occurrence during the simulation.
